# Oestrogen and parathyroid hormone alleviate lumbar intervertebral disc degeneration in ovariectomized rats and enhance Wnt/β-catenin pathway activity

**DOI:** 10.1038/srep27521

**Published:** 2016-06-09

**Authors:** Haobo Jia, Jianxiong Ma, Jianwei Lv, Xinlong Ma, Weiguo Xu, Yang Yang, Aixian Tian, Ying Wang, Lei Sun, Liyan Xu, Lin Fu, Jie Zhao

**Affiliations:** 1 Department of Orthopedics Institute, Tianjin Hospital, 406, Jiefang Nan Street, Hexi District, Tianjin 300211, China; 2 Biomechanics Labs of Orthopedic Research Institute, Tianjin Hospital Heping Branch, 122, MuNan Street, Heping District, Tianjin 300050, China; 3 Tianjin Medical University, No. 22, Qixiangtai Road, Heping District, Tianjin 300070, China

## Abstract

To investigate the mitigation effect and mechanism of oestrogen and PTH on disc degeneration in rats after ovariectomy, as well as on Wnt/β-catenin pathway activity, thirty 3-month-old rats were ovariectomized and divided into three groups. Ten additional rats were used as controls. Eight weeks later, the rats were administered oestrogen or PTH for 12 weeks, and then discs were collected for tests. Results showed that nucleus pulposus cells in the Sham group were mostly notochord cells, while in the OVX group, cells gradually developed into chondrocyte-like cells. Oestrogen or PTH could partly recover the notochord cell number. After ovariectomy, the endplate roughened and endplate porosity decreased. After oestrogen or PTH treatment, the smoothness and porosity of endplate recovered. Compared with the Sham group, Aggrecan, Col2a and Wnt/β-catenin pathway expression in OVX group decreased, and either oestrogen or PTH treatment improved their expression. The biomechanical properties of intervertebral disc significantly changed after ovariectomy, and oestrogen or PTH treatment partly recovered them. Disc degeneration occurred with low oestrogen, and the underlying mechanisms involve nutrition supply disorders, cell type changes and decreased Wnt/β-catenin pathway activity. Oestrogen and PTH can retard disc degeneration in OVX rats and enhance Wnt/β-catenin pathway activity in nucleus pulposus.

Due to a rapid drop in oestrogen levels, tissue and system metabolism may change in postmenopausal women, and postmenopausal osteoporosis gradually develops. Clinical studies reported that the incidence and severity of disc degeneration in elderly women were higher than those in age-matched men, which indicates that disc degeneration displays gender differences^1^,^2^,^3^. The intervertebral disc consists of nucleus pulposus (NP), annulus fibrosus (AF) and cartilage endplate, and together, they determine the disc state^4^. NP cells originate from notochord cells, and the life span of notochord cells is different among different species. In pigs, rabbits, rodents and non-chondrodystrophoid dogs, cells in the NP are mainly notochord cells; however, in humans, sheep and chondrodystrophoid dog breeds (CDBs, such as beagle dog), notochord cells gradually disappear and are replaced by chondrocyte-like cells^5^,^6^,^7^,^8^. Notochord cells have the proliferative ability to maintain a healthy nucleus pulposus and prevent degeneration, while chondrocyte-like cells are terminally differentiated cells with no proliferative capacity and lower matrix-forming ability^5^,^8^,^9^. Therefore, the proportion of notochord cells and chondrocyte-like cells in the NP can reflect the degree of degeneration.

The endplate contains marrow contact channels (MCC), which provide nutrients for the intervertebral disc. The intervertebral disc is a non-vascular structure in which metabolic wastes and nutrients exchange mainly through cartilage endplate by diffusion and liquid flow, and calcification of the cartilage endplate may accelerate disc degeneration^10^,^11^,^12^,^13^. A finite element study showed that endplate stress would increase when the bone mass of the vertebral body decreased, and abnormal stress could lead to endplate calcification and obstruction of marrow contact channels, thus giving rise to disc degeneration^14^.

In view of the fact that nutrient molecules can enter the NP through the endplate, oestrogen and other hormones are also likely to enter the NP and have direct effects on the growth and metabolism of NP cells. The matrix of the intervertebral disc is mainly composed of Aggrecan, collagen and elastin, and oestrogen and parathyroid hormone (PTH) have an important role in promoting their metabolism^15^,^16^. In addition, oestrogen receptors α and β were found in both the NP and the AF, and experiments confirmed that oestrogen can directly improve the proliferative activity of cells in the NP and annulus fibrosus and reverse their apoptosis^17^,^18^,^19^. PTH can also enhance matrix synthesis of NP and AF cells and reduce calcification^16^.

The Wnt/β-catenin pathway plays an important role in development and growth of the intervertebral disc, especially for notochord cells^20^,^21^. Disc degeneration occurs very early in chondrodystrophoid dogs, with notochord cells in the NP gradually disappearing and being replaced by chondrocyte-like cells, and the process is accompanied by reduced Wnt/β-catenin pathway activity. After ovariectomy, osteoporosis is induced, and disc degeneration may also occur. However, changes in the Wnt/β-catenin pathway during the process have not been reported^4^. Oestrogen and PTH can both act directly on the intervertebral disc, and their effects on the Wnt/β-catenin pathway in ovariectomized rats remain unclear^16^,^22^,^23^. In the present study, we evaluated disc degeneration in ovariectomized rats, the ability of oestrogen or PTH to alleviate these effects and changes in the Wnt/β-catenin pathway during the process.

## Materials and Methods

### Experimental design

Forty three-month-old virgin female SD rats were randomly divided into four groups (n = 10 per group). Three groups underwent ovariectomy surgery and were left untreated for 8 weeks. The fourth group underwent a sham operation (Sham Group). The OVX+E2 group was subcutaneously injected with 17β-estradiol (Sigma-Aldrich, St. Louis, MO, USA) at a dose of 25 μg/kg five times a week. The OVX+PTH group was subcutaneously injected with 80 μg/kg synthetic human PTH(1–34) (Mimotopes Peptides company, Melbourne, VIC, Australia) five times a week. The sham group and the OVX group were treated with vehicle. Drug intervention was conducted for 12 weeks; then, the rats were sacrificed, and intervertebral discs or spinal motion segments were harvested. All procedures were approved by the Ethics Committee of Tianjin Hospital and performed according to the Guide for Care and Use of Laboratory Animals.

### Histological evaluation

After fixation with paraformaldehyde solution and decalcification, L5-L6 segments were sliced along the median sagittal plane. After serial dewaxing, sections were stained with haematoxylin and rinsed with distilled water. Then, the sections were differentiated in hydrochloric acid alcohol and stained with 0.5% eosin. The sections were observed with a Digital Image Analyzer (Ni-E, Nikon, Japan), and the degree of disc degeneration was scored according to the following scoring system: NP:0, abundant notochordal cells; 1, notochordal cells loss; chondrocyte-like cells emergence; 2, focal mucoid degeneration; 3, diffuse mucoid degeneration and clefts. AF: 0, compact lamellae; 1, proliferation of fibrocartilaginous tissue and loss of nuclear–annular border; 2, emergence of fissures. Osteophyte: 0, absence; 1, appearance; 2, overgrowth^4^.

### Evaluation of endplate degeneration

L2 vertebral bodies were scanned by Siemens Micro-CT scanning system. The scan voltage was 80 kV, the current was 500 μA and the layer spacing was 8.5 μm. The endplates were evaluated according to surface smoothness and features of the margin^24^. Superior endplates were reconstructed, and volume ratios of marrow contact channels in the endplate (porosity) were calculated to estimate the condition of endplate nutritional supply and indirectly reflect the state of the endplate.

### PCR analysis

After the rats were sacrificed, the T12/L1 and L1/2 intervertebral discs were immediately immersed in RNAstore reagent and frozen in liquid nitrogen. Total RNA was extracted using TriPure Isolation Reagent (Roche Diagnostics, Indianapolis, IN, USA), and the concentration and purity were measured.RNA (1 μg) was reverse-transcribed using a First-Strand cDNA Synthesis kit (GeneCopoeia, Guangzhou, China). The transcripts of interest and of the housekeeping gene β-actin were amplified from first-strand cDNA by real-time PCR.

### Immunohistochemistry

The L5-L6 segments were sliced along the median sagittal plane. After pre-treatment with 5% BAS blocking solution, sections were incubated in diluted antibody (dilution ratio: Coll2 1: 150; MMP-3 1: 120; Aggrecan 1: 150; β-catenin 1: 100) (Boster, Wuhan, China) separately at 4 °C overnight. Then, sections were incubated at 37 °C for 30 min with biotin-coupled secondary antibody diluted 1:150 and SABC. DAB colouration solution was formulated and dropped on sections and sections were observed with a Digital Image Analyzer. All sections were semi-quantitatively analyzed by Image-Pro Plus 6.0 software, and the average optical density was measured on the images at 400× magnification.

### Western blot analysis

Nucleoprotein was isolated from nucleus pulposus lysates of L2/3 and L4/5 discs using a Nuclear Extract Kit (Active Motif, Carlsbad, CA). Protein (100 μg) was separated by electrophoresis in a 10% polyacrylamide separating gel and transferred to an Immobilon PVDF membrane (Millipore, Bedford, MA, USA). The membrane was blocked with 5% BSA TBST blocking buffer for 1 h and then incubated with rabbit polyclonal anti-mouse β-catenin antibody (1:200 in 5% BSA TBST buffer) for 2 h and goat anti-rabbit secondary antibody (1:1000) for 1 h at room temperature. ECL chemiluminescence solution was uniformly dropped on the transferring membrane, and the results were recorded using a chemiluminescence imaging system.

### Biomechanical tests

**Figure 1:**
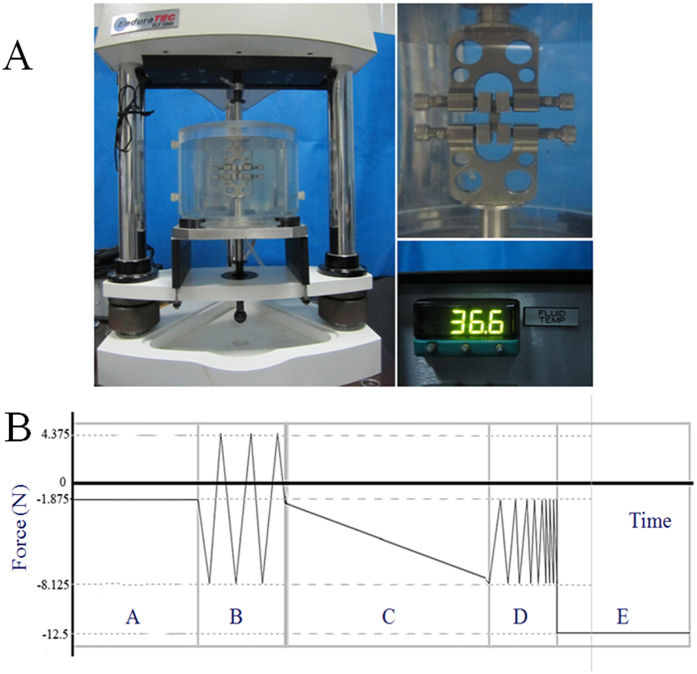
Biomechanical tests of the intervertebral disc. (**A**) Specimen fixation and temperature control; (**B**) mechanical protocol: A) equilibration, B) cyclic compression-tension, C) quasi-static compression, D) frequency sweep and E) creep.

The most important function of the intervertebral disc is to conduct loads and absorb shocks; thus, one of the most accurate assessments of disc degeneration is to evaluate its biomechanical properties. In the present study, L3-L4 segments were subjected to biomechanical tests to assess changes in their biomechanical properties after ovariectomies and drug intervention. The L3-L4 motion segments were dissected, and then posterior elements were removed and fixed in an Enduratec ELF 3200 custom clamp in a PBS solution, as shown in Fig. 1A^25^. The biomechanical tests consisted of five force-controlled loading mode phases: A) equilibration, B) cyclic compression-tension, C) quasi-static compression, D) frequency sweep and E) creep^26^: Stage A consisted of a 30-min dwell at −1.875 N; Stage B included sinusoidal tension-compression for 20 cycles at 1 Hz with a 6.25 N amplitude centred at approximately-1.875 N; Stage C subjected specimens to a slow compressive ramp at 0.005 N/s from −1.875 to −8.125 N; Stage D consisted of five cycles at five different frequencies: 0.05, 0.1, 0.5, 1, and 5 Hz applied from −1.875 to −8.125 N in sinusoidal compression; Stage E consisted of a 12.5 N dwell for 30 min. The code editor and curve fitting tool of MATLAB (Mathworks) were used for fitting analysis, and corresponding mechanical parameters were calculated according to formula recommended by Barbir^26^. For stages A and E, the creep displacement, d, was fit to a stretched exponential function


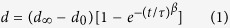


where d_inf_ –d_0_ is the height loss of equilibrium, t is time, τ is a time constant and β is a stretch constant. In stage B, the neutral zone length and three dynamic stiffness values (compression, neutral zone and tension) were calculated using a trilinear fit. In stage C, the quasi-static stiffness was calculated by linear regression of force–displacement data. The dynamic stiffness (K*) was calculated in stage D.


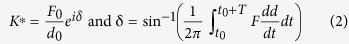


where F is force, d_0_ is displacement, T is period and t is time^26^.

### Statistical analyses

All data are presented as the mean ± SD. Significant differences were determined by one-way ANOVA followed by Dunnett’s test for post hoc comparisons or non-parametric Kruskal–Wallis test to identify significant pair-wise differences, with *p* < 0.05 considered to be significant. GraphPad PrismV5.01 was used for statistical analyses.

## Results

### Histological evaluation

**Figure 2:**
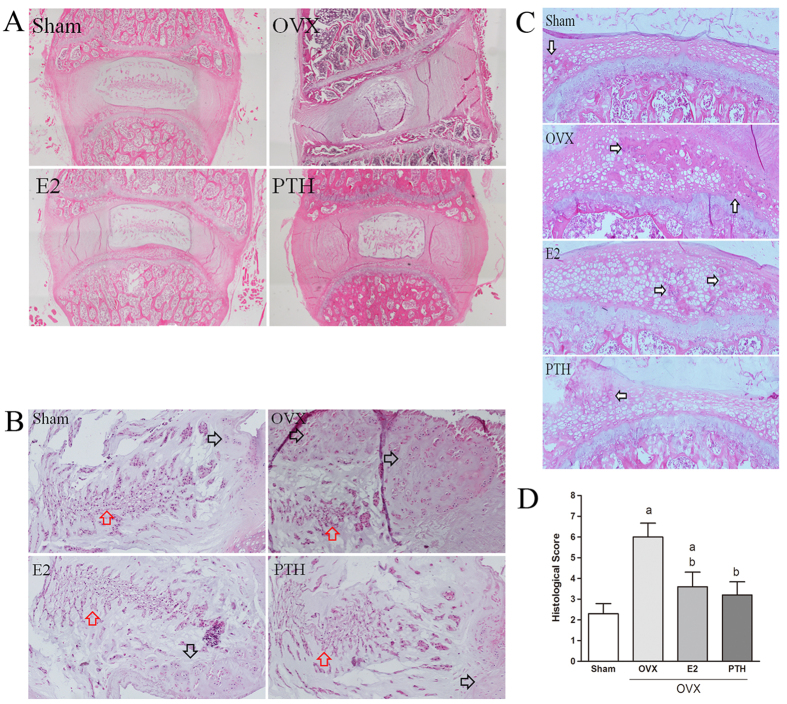
(**A**) Histological illustration of the L5–L6 segments 5 months post-surgery; (**B**) Histological illustration of the nucleus pulposus (Red arrows: notochord cells; Black arrows: chondrocyte-like cells); (**C**) Ectopic bone tissue in cartilage endplate of each group; (**D**) Histological score of disc degeneration of the four groups (^a^p < 0.05 compared with the Sham group; ^b^p < 0.05 compared with the OVX group).

Histological evaluation of intervertebral discs is shown in Fig. 2A–C. In the Sham group, NP cells were mostly notochord cells, and only a small number of chondrocyte-like cells emerged at the NP edge. The AF structure was intact, collagen fibres were arranged tightly, and there were clear boundaries between the AF and the NP. Cells in the cartilage endplate were arranged regularly, with only a small amount of ectopic bone tissue. In the OVX group, the number of notochord cells in the NP was significantly reduced, and many clustered chondrocyte-like cells appeared, surrounded by a mucoid degenerated matrix. Fissures appeared in the AF, collagen fibres were disorganized, and proliferation of fibrocartilage emerged, making the boundary between the AF and NP unclear. Ectopic bone tissue appeared in cartilage endplate. Compared with the OVX group, in the OVX+E2 group, the number of chondrocyte-like cells was significantly reduced, and the matrix was similar to that observed in Sham group, with no regions of large mucoid degeneration. Collagen fibres were arranged more regularly, and the amount of ectopic bone tissue was significantly reduced. Histological evaluation of the OVX+PTH group was similar to that of the OVX+E2 group. NP cells were mainly notochord cells, with no mucoid degeneration in the matrix. Only a few fissures could be seen in the AF, and collagen fibres were arranged regularly. A very small amount of ectopic bone tissue could be seen in the cartilage endplate.

Based on the assessment using the scoring system, the intervertebral discs of the Sham group were in good condition, and histological scores were low (2.3 ± 0.48). The intervertebral discs of the OVX group had degenerated significantly, and the histological scores were 6.0 ± 0.67, which were higher than those in the other three groups (*p* < 0.05). After 17β-estradiol treatment, disc degeneration was significantly alleviated, and the scores were 3.6 ± 0.69 and were lower than those of the OVX group and higher than those of the Sham group (*p* < 0.05). PTH exerted a stronger effect than oestrogen (3.2 ± 0.63 VS 3.6 ± 0.69, *p* < 0.05).

### Evaluation of endplate degeneration

**Figure 3:**
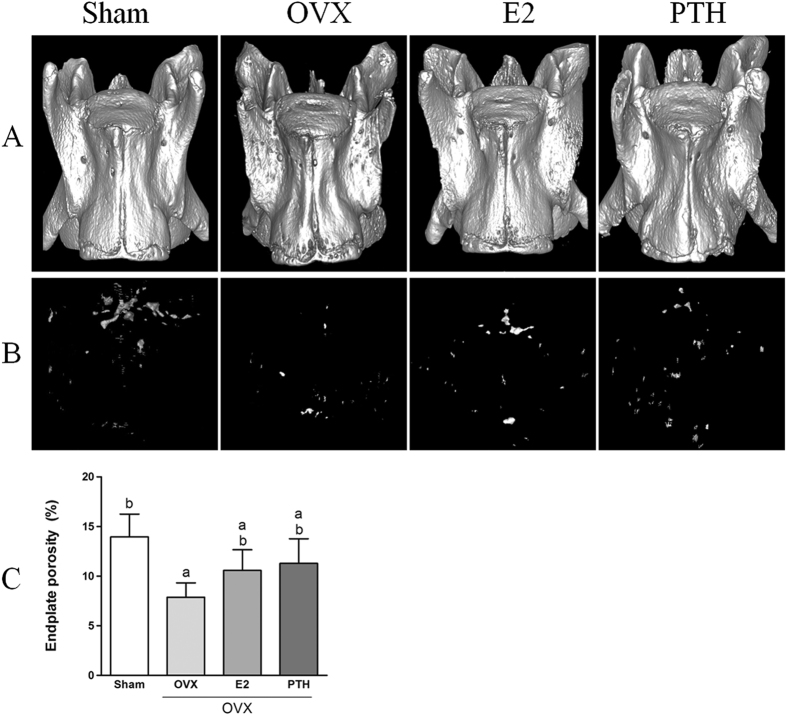
(**A**) Three-dimensional reconstruction of L2 in each group. (**B**) Three-dimensional reconstruction of marrow contact channels. (**C**) Endplate porosity of L2 superior endplates (^a^p < 0.05 compared with the Sham group; ^b^p < 0.05 compared with the OVX group).

As shown in Fig. 3A, compared with the Sham group, small pits appeared on endplate surfaces in the OVX group, the surfaces became rough, and the boundary with the vertebral body became indistinct, indicating that the endplate gradually degenerated after ovariectomy. After oestrogen or PTH therapy, the smoothness of endplate surface recovered, and margin irregularity improved, indicating that both oestrogen and PTH improved the state of the endplate.

Three-dimensional reconstruction of MCC in L2 superior endplates and porosity calculation results are shown in Fig. 3B,C. The average porosity in the Sham group was 13.96%. The number and volume of MCC in OVX rats were significantly reduced, with the porosity decreasing to 7.88% (*p* < 0.05). These results indicate that ossification occurred and the disc nutrient supply pathway was blocked. In the OVX+E2 group and the OVX+PTH group, both the number and volume of MCC increased, with the porosity improving to 10.57% and 11.29%, respectively, which was higher than that in the OVX group (*p* < 0.05) but had not reached the level observed in the Sham group (*p* < 0.05).

### RT-PCR results

**Figure 4:**
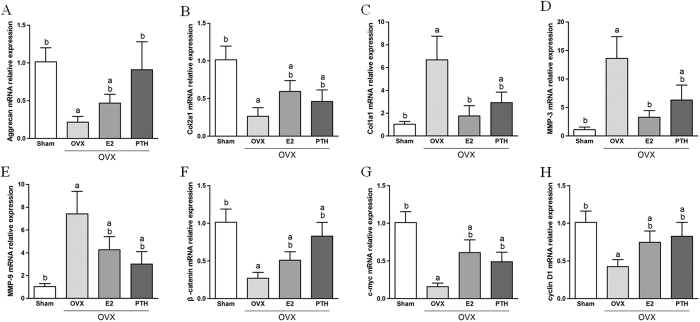
mRNA expression of disc components and the Wnt/β-catenin pathway: (**A**) Aggrecan, (**B**) Col2α1,(**C**) Col1α1, (**D**) MMP-3, (**E**) MMP-9, (**F**) β-catenin, (**G**) c-myc and (**H**) cyclin D1 (^a^p < 0.05 compared with the Sham group; ^b^p < 0.05 compared with the OVX group).

As shown in Fig. 4, compared with the Sham group, Aggrecan and Col2a1 mRNA expression decreased and Col1a1 mRNA expression increased at five months post ovariectomy (*p* < 0.05). After oestrogen or PTH therapy, Aggrecan and Col2a1 mRNA expression increased and Col1a1 mRNA expression decreased, and the differences with the OVX group were statistically significant (*p* < 0.05).

For MMPs, which can degrade intervertebral disc matrix, MMP-3 and MMP-9 mRNA expression significantly increased in the OVX group compared with the Sham group (*p* < 0.05). MMP-3 and MMP-9 mRNA expression in both the OVX+E2 and OVX+PTH groups were lower than those in the OVX group (*p* < 0.05).

In the Wnt/β-catenin pathway, β-catenin, c-myc and cyclin D1 mRNA expression decreased in OVX rats (*p* < 0.05), indicating that Wnt/β-catenin pathway down-regulation accompanied disc degeneration caused by ovariectomy. β-catenin, c-myc and cyclin D1 mRNA expression in the OVX+E2 and OVX+PTH groups were higher than those in the OVX group, indicating that oestrogen and PTH could increase the Wnt/β-catenin pathway expression at the same time as when they relieve disc degeneration.

### Immunohistochemistry

**Figure 5:**
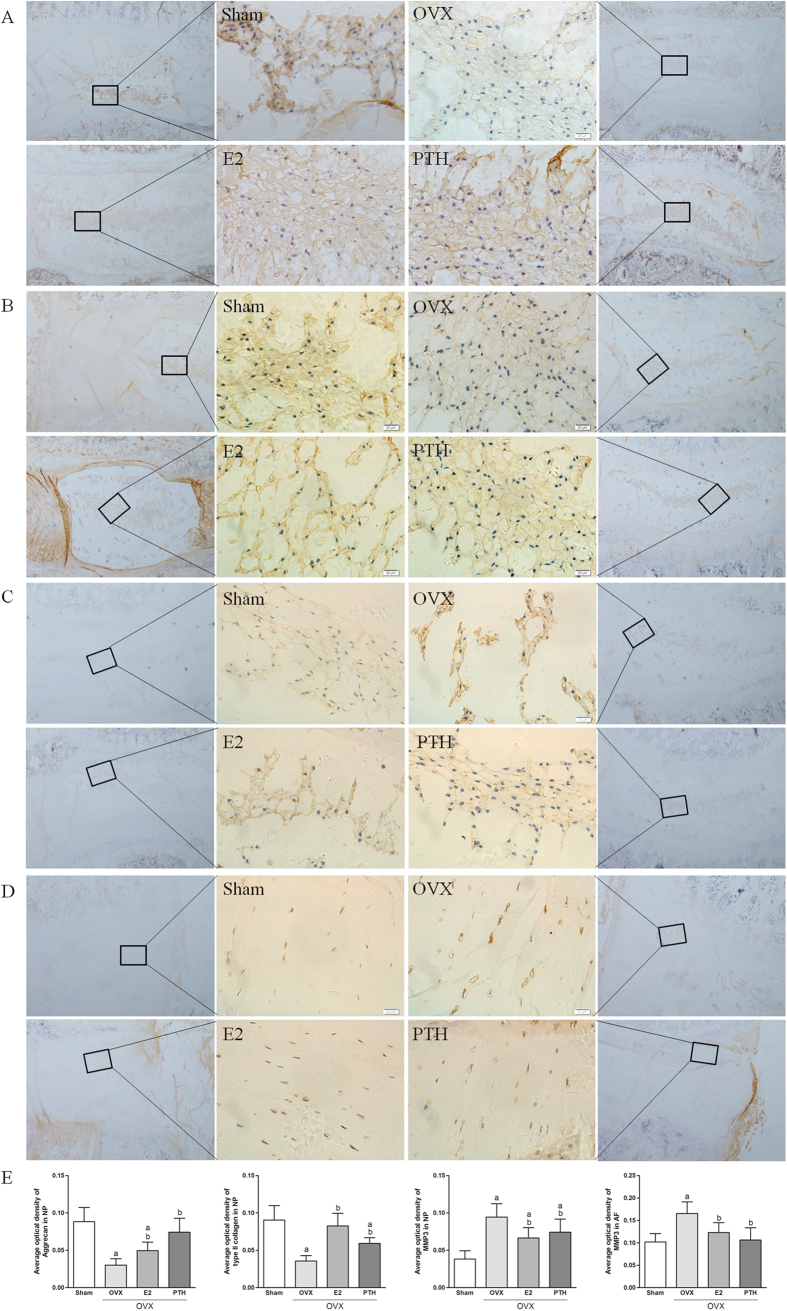
Immunohistochemical staining of the nucleus pulposus ((**A**) Aggrecan; (**B**) type II collagen and (**C**) MMP-3) and annulus fibrosis ((**D**) MMP-3), and the average optical density of them (**E**) (^a^p < 0.05 compared with the Sham group; ^b^p < 0.05 compared with the OVX group).

Immunohistochemical staining of the NP (Fig. 5A–C) showed that Aggrecan and type II collagen contents were high and the MMP-3 content was low in the Sham group, indicating that the NP was in a healthy state. In the OVX group, Aggrecan and type II collagen staining intensities were significantly lower and the MMP-3 staining intensity was higher than those of the Sham group. After oestrogen or PTH intervention, Aggrecan and type II collagen staining intensities increased and MMP-3 staining intensity decreased compared with the OVX group. Immunohistochemical staining of MMP-3 in annulus fibrosis (Fig. 5D) showed that in the OVX group, the staining intensity of MMP-3 was higher compared to the Sham group. Compared with the OVX group, the staining intensity of MMP-3 in both the OVX+E2 group and the OVX+PTH group decreased. The average optical density of them in each group was shown in Fig. 5E. Immunohistochemistry results showed that oestrogen and PTH could improve the expression of normal disc components and inhibit enzymes that can degrade the matrix.

### Immunohistochemistry and western blot of β-catenin in NP

**Figure 6:**
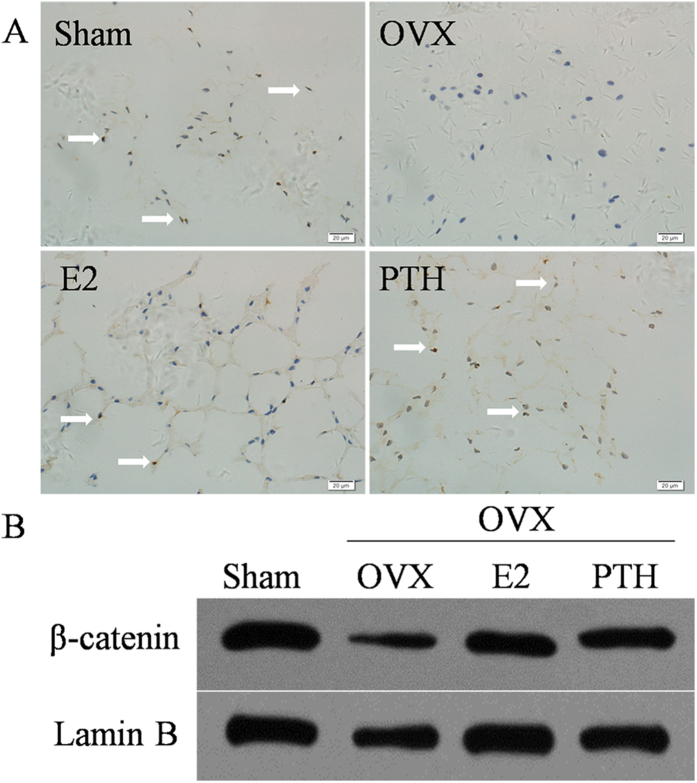
Immunohistochemistry (**A**) and western blot (**B**) of β-catenin in the nucleus pulposus.

The results showed that immunohistochemical staining of β-catenin could be observed in NP cells in the Sham group, indicating that there was a certain degree of expression of the Wnt/β-catenin pathway in the normal disc, as shown in Fig. 6. In the OVX group, notochord cells developed into chondrocyte-like cells, and the β-catenin staining intensity in the NP was lower compared to the Sham group. Compared with the OVX group, in the OVX+E2 group and the OVX+PTH group, the number of notochord cells increased and the number of chondrocyte-like cells decreased significantly, accompanied by an increased β-catenin staining intensity in the NP. The Western blot of nuclear β-catenin was consistent with the immunohistochemistry results.

### Biomechanical properties

**Figure 7:**
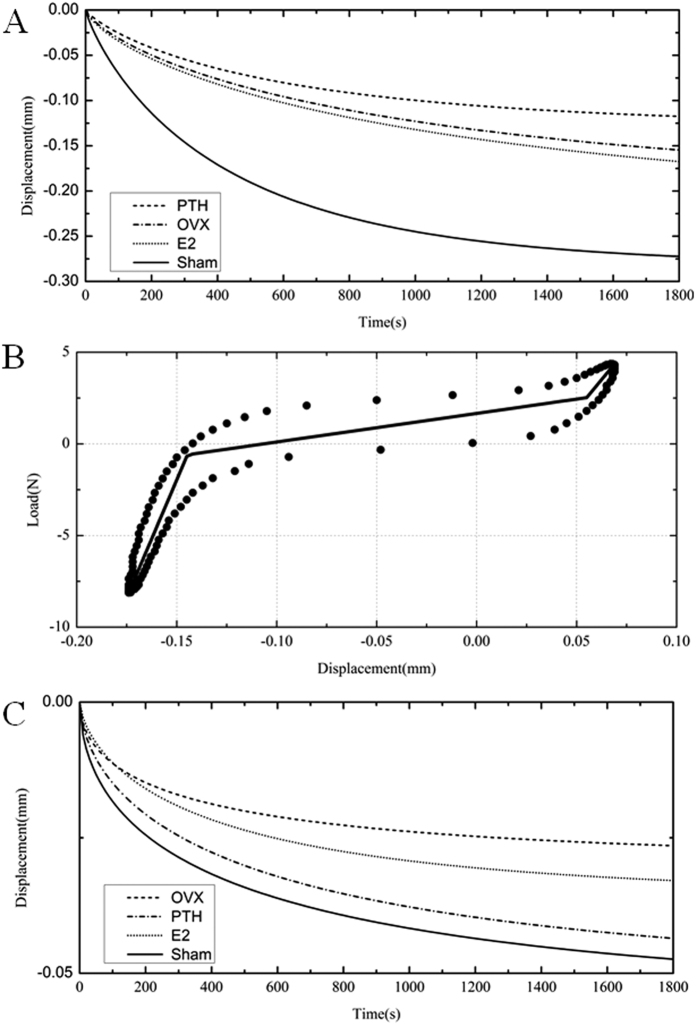
(**A**) Transient deformation patterns of equilibration (stage A) for simulated stretched exponential fitting using the mean parameters for each experimental group. (**B**) Trilinear fits to data from the compression-tension conditioning stage of a sample from theSham group. (**C**) Transient deformation patterns of creep (stage E) for simulated stretched exponential fitting.

The biomechanical properties of the intervertebral disc significantly changed after ovariectomy or oestrogen and PTH treatment. The biomechanical features of each stage are shown in Table 1. In stage D, no significant effects of frequency were detected, and thus, dynamic stiffness was presented with the 1 Hz data. In stage A, the equilibrium height loss (d_inf_ –d_0_) of the OVX group decreased by 54% compared with that of the Sham group, and 12 weeks of oestrogen or PTH treatment resulted in a 77% or 53% increase compared with OVX rats, respectively (*p* < 0.05). Deformation fitting curves under sustained −1.875 N in stage A of the four groups are shown in Fig. 7A.

Trilinear fitting was used to calculate the dynamic stiffness values in stage B, and one fitting result of the Sham group is shown in Fig. 7B. The compressive stiffness, tensile stiffness and neutral zone stiffness increased and the neutral zone length decreased at 5 months post-surgery, indicating degeneration of biomechanical properties of the intervertebral disc. Compared with the OVX group, the three dynamic stiffness values decreased and the neutral zone length increased in both the OVX+E2 group and the OVX+PTH group, suggesting that oestrogen or PTH treatment could recover their biomechanical properties.

Quasi-static compression was used to evaluate static mechanical properties of the intervertebral disc. Twenty weeks of low oestrogen levels resulted in a 53% increase in static stiffness compared with Sham rats, while oestrogen or PTH therapy reduced quasi-static stiffness by 23% or 20% compared with OVX rats, respectively (*p* < 0.05). A dynamic frequency sweep test (stage D) reflects disc mechanical properties under dynamic loading. At specified frequencies, the dynamic stiffness of the OVX group increased by 46% compared to that of the Sham group, while the dynamic stiffness values of the OVX+E2 group and the OVX+PTH group were 86% and 82% of that of the OVX group, respectively (*p* < 0.05).

Deformation fitting curves of the four groups under sustained −12.5 N in a creep test (stage E) are shown in Fig. 7C. Compared with the Sham group, creep height loss (d_inf_ –d_0_) in ovariectomized rats decreased to46% (*p* < 0.05), indicating that the viscoelasticity of the intervertebral disc decreased and that the disc degenerated. Oestrogen treatment increased the creep height loss by 20% compared with the OVX group, but the differences were not statistically significant (*p* > 0.05). PTH recovered the creep height loss to 93% of that in the Sham group (*p* > 0.05), which significantly improved disc viscoelasticity. Consistent with the trend of creep height loss (d_inf_ –d_0_), the cumulative height loss in both the OVX and OVX+E2 groups was lower than that in the Sham group (*p* < 0.05). In stage E, the cumulative height loss of the OVX+PTH group recovered to 82% of that of the Sham group (*p* < 0.05).

## Discussion

### Role of endplate in disc degeneration caused by ovariectomy

Disc degeneration is a common clinical disease, but the initiating and influencing factors remain unclear^27^. It has been reported that disc degeneration and disc space narrowing are more severe in female patients than in male patients, indicating that disc degeneration displays significant gender differences^1^,^2^,^3^,^28^. Those findings suggested that sex hormones, especially oestrogen, may play an important role in disc degeneration. In postmenopausal women, oestrogen levels drop rapidly, and this may promote disc height reduction, while oestrogen replacement therapy restores disc height^23^,^29^,^30^. The intervertebral disc is composed of inner highly hydrated NP and peripheral annulus fibrosis. The inner layers of the annulus fibrosis have a slightly higher degree of hydration, while the outer layers have a lower degree of hydration, but with tightly combined fibres^31^. Peripheral micro-circulatory system can only provide a part of the outer layers with nutrients, and nutrients for the NP and inner layers are supplied from bone marrow channels (capillaries or medullary sinuses) inside the endplate; thus, the endplate is the most important nutrient supply channel of the disc^32^. When calcification occurs in the endplate, the number and volume of capillaries or medullary sinuses decreases and nutrient supply to NP decreases, thus accelerating disc degeneration^33^. Drastically reduced oestrogen levels cause vertebral osteoporosis, perfusion of vertebral bone marrow and reduced MCC in the endplate, which directly causes a reduced disc nutrient supply and ultimately leads to disc degeneration^34^. In addition, abnormal stress can also lead to cartilage endplate calcification, thereby causing disc degeneration^34^. A finite element study found that with an osteoporotic vertebral body, the peak pressure of the cartilage endplate would increase and the abnormal stress would promote its calcification^14^. In the present study, Micro-CT was used to show that the number and volume of MCC decreased and ossification occurred in the endplate after ovariectomy. In addition, Micro-CT technology allowed us to observe the endplate surface and margin in a three-dimensional view. Endplate surface degeneration also had an adverse impact on the disc nutritional supply, and it was difficult to obtain those results with a two-dimensional histological study. Oestrogen and PTH could alleviate spinal osteoporosis in ovariectomized rats and increase MCC volume in the endplate, thus improving disc nutrition supply and alleviating disc degeneration.

### The effect of oestrogen and PTH on disc components

In addition to the effects discussed above, oestrogen directly promotes collagen metabolism and biochemical function of proteoglycans^35^,^36^. Though the intervertebral disc is avascular, in view of the fact that nutrients can enter the nucleus pulposus through the endplate, oestrogen and other hormones can potentially also enter the nucleus pulposus and play a direct role in the growth and metabolism of NP cells. Studies have demonstrated that oestrogen receptors α and β exist in the NP and AF and have shown that oestrogen can directly improve proliferative activity of NP and AF cells, which suggested that oestrogen could play a regulatory role in the disc through oestrogen receptors *in vivo*^17^,^18^,^19^. The intervertebral disc contains a variety of collagens and proteoglycans, with type II collagen and Aggrecan being the most important types. Type II collagen plays a very important role in maintaining NP biomechanical function and Aggrecan is responsible for maintaining osmotic pressure and hydration of the intradiscal matrix^37^,^38^,^39^,^40^. In OVX rats, positive regulation of oestrogen decreased and synthesis of collagen, proteoglycan and other matrix proteins decreased. Scholars have also demonstrated that PTH can promote synthesis of the cellular matrix in the disc and suppress disc calcification^16^. The present study showed that after ovariectomy, mRNA and protein expression of Aggrecan and type II collagen in the disc decreased, while type I collagen, MMP-3 and MMP-9 expression increased. Compared with the OVX group, mRNA and protein levels of Aggrecan and type II collagen in the OVX+E2 and OVX+PTH groups were higher, and expression of type I collagen, MMP-3 and MMP-9 were lower, showing that oestrogen and PTH could improve disc matrix synthesis and inhibit enzymes that can degrade extracellular matrix to reverse disc degeneration caused by ovariectomy.

### Effect of oestrogen and PTH on Wnt/β-catenin pathway of NP in OVX rats

Nucleus pulposus plays a central role in maintaining the healthy state and function of the intervertebral disc, and many studies on disc degeneration focused on NP cells object^41^,^42^. Although early processes in disc degeneration have been investigated in histopathology, the biomolecular signal transduction pathways involved in the conversion of notochord cells into chondrocyte-like cells remain unclear^43^,^44^. Among the many signalling pathways, the Wnt/β-catenin pathway plays an important regulatory role in disc embryonic development and has become a hot spot in disc degeneration in recent years^20^,^45^,^46^. Smolders and his colleagues used Dual channel DNA microarrays to compare healthy NP containing only notochord cells (notochord cells-rich), NP with a mixed population of notochord cells and chondrocyte-like cells (Mixed) and NP containing solely chondrocyte-like cells (chondrocyte-like cells-rich) in both non-chondrodystrophic and chondrodystrophic dogs^47^. They found that compared with notochord cell-rich and mixed groups, Axin2 (a marker of Wnt/β-catenin pathway activity) expression in the chondrocyte-like cell-rich group was significantly reduced. Subgroup analysis of the three different degeneration degrees showed that axin2 gene expression was significantly higher in chondrodystrophic dogs compared with non-chondrodystrophic dogs. Their findings showed that at the early stage of disc degeneration in dogs, the number of notochord cells decreased, and the number of chondrocyte-like cells increased, along with reduced expression of Wnt/β-catenin, which was consistent with the results of the present and previous studies^45^,^46^. However, some studies reported that compared with healthy controls, Wnt/β-catenin pathway activity in a degenerated intervertebral disc was up-regulated^48^,^49^,^50^. The two conclusions seem contradictory, and the reason for this discrepancy may be that the cell types and stages of disc degeneration were different between the studies. In Smolders’ research, the degeneration was in the early stage, and notochord cells were gradually replaced by chondrocyte-like cells, where as NP cells in adult humans were mostly chondrocyte-like cells, and disc degeneration was in the late stage. In SD rats, NP cells are mostly notochord cells, and disc degeneration emerged after ovariectomy, accompanied by gradual development of notochord cells into chondrocyte-like cells. Our results showed that during the process of disc degeneration caused by ovariectomy, expression levels of β-catenin, c-myc and cyclin D1 all decreased, indicating that Wnt/β-catenin pathway activity decreased. Oestrogen and PTH increased the proportion of notochord cells in the nucleus pulposus of OVX rats and increased the expression of β-catenin, c-myc and cyclin D1 at the same time.

The mechanisms of how ovariectomy or oestrogen and PTH regulate the Wnt/β-catenin pathway in a disc are complicated and remain unclear. In contrast to the intervertebral disc, cell types in the bone remain unchanged, and oestrogen and PTH regulate osteoblast or osteocytes metabolism through various mechanisms, for example, by reducing the Wnt/β-catenin pathway inhibitor DKK1 or SOST^51^,^52^. In the intervertebral disc of SD rats, oestrogen levels drop quickly after ovariectomy, and the direct regulatory effect of oestrogen on NP cells decreased. In addition, postmenopausal osteoporosis developed, and thus, the stress load on the disc increased and the nutrition supply to the disc decreased. Under the combined effect of the three above-mentioned factors, the intervertebral disc gradually degenerated and cell types changed, along with a decrease in Wnt/β-catenin pathway activity. One possible mechanism is that oestrogen or PTH has a direct regulatory effect on NP cells via oestrogen receptors (α and β) or the PTH receptor (and PTHrP) by regulating key proteins of the Wnt/β-catenin pathway such as Axin2 or GSK-3β or by reducing the Wnt/β-catenin pathway inhibitors DKK-1 or SOST to indirectly maintain and improve the Wnt/β-catenin pathway in NP cells^53^. Another possible mechanism is that the basic expression levels of the Wnt/β-catenin pathway are different between notochord cells and chondrocyte-like cells, which are similar to osteoblasts and chondrocytes. Osteoblasts and chondrocytes both originate from bone marrow-derived mesenchymal stem cells (BMSCs) and have many similar features, but the basic expression levels of Wnt/β-catenin pathway components are different. In osteoblasts, Wnt/β-catenin pathway components are highly expressed to maintain bone formation activity and the normal state of osteoblasts, while in chondrocytes, increased expression of Wnt/β-catenin pathway components indicates degeneration, as has been shown in osteoarthritis^54^. For notochord cells and chondrocyte-like cells, the Wnt/β-catenin pathway is highly expressed in notochord cells to maintain proliferative activity and matrix production capacity, while the Wnt/β-catenin pathway is in a low expression state in chondrocyte-like cells and high expression level of Wnt/β-catenin pathway indicates degeneration. After SD rats were ovariectomized, disc degeneration occurred, the number of notochord cells decreased, the number of chondrocyte-like cells increased, and in general, the Wnt/β-catenin pathway activity decreased. Oestrogen and PTH alleviated disc degeneration via the above three factors and improved the proportion of notochord cells and the activity of the Wnt/β-catenin pathway. Regulating mechanisms of the Wnt/β-catenin pathway in the disc are very complicated, and research has only recently started to focus on these mechanisms and has reported conflicting conclusions^45^,^46^,^47^,^49^,^50^,^55^. The present study was performed with female SD rats; the intervention included ovariectomy and oestrogen or PTH replacement, the degeneration time was set to 5 months after ovariectomy, and we studied the Wnt/β-catenin pathway changes during disc degeneration. After collecting the preliminary results, we will conduct more in-depth studies to reveal the specific mechanisms.

Our study has some limitations. The present study explored the effect of oestrogen and PTH on the Wnt/β-catenin pathway in intervertebral discs for the first time. Thus, only one time point was chosen. Investigation of dynamic changes of disc degeneration and the Wnt/β-catenin pathway will allow us to better understand the effects. In future studies, more time points will be included to explore the dynamic effects and long-term treatment effects on disc degeneration and the Wnt/β-catenin pathway. In addition, to strengthen our conclusion, additional studies need to be performed, such as treating the OVX rats with Wnt3a to determine whether it can prevent OVX-induced disc degeneration and treating the OVX rats with Wnt/β-catenin pathway inhibitor XAV-939 to determine whether it can reduce the therapeutic effect of oestrogen or PTH. Finally, as the first in a series of studies on disc degeneration, the present study was just an intervention study on the animal level, and subsequent studies will include cell experiments to study the in-depth relationship of the nucleus pulposus cells and the Wnt/β-catenin pathway.

In general, disc degeneration develops with low oestrogen levels, and the underlying mechanisms involve nutrient supply disorders, cell type changes (a decrease in notochord cells and an increase in chondrocyte-like cells), matrix degradation and reduced Wnt/β-catenin pathway activity. Oestrogen and PTH treatment can retard the progression of lumbar intervertebral disc degeneration in OVX rats, improve the proportion of notochord cells and enhance Wnt/β-catenin pathway activity in the NP. The mechanisms of how ovariectomy or oestrogen and PTH regulate the Wnt/β-catenin pathway in the disc are complicated and remain unclear. After collecting the preliminary results, we will next conduct more in-depth studies to reveal the specific mechanisms.

## Additional Information

**How to cite this article**: Jia, H. *et al*. Oestrogen and parathyroid hormone alleviate lumbar intervertebral disc degeneration in ovariectomized rats and enhance Wnt/β-catenin pathway activity. *Sci. Rep.* **6**, 27521; doi: 10.1038/srep27521 (2016).

## Figures and Tables

**Table 1:** Summary of mechanical parameters (Mean ± SD) calculated at each testing stage of each group.

Test Stage	Mechanical parameter	Sham group	OVX group	OVX+E2group	OVX+PTH group
A. Equilibration	d_inf_ –d_0_ (mm)	0.28 (0.05)^b^	0.13 (0.04)^a^	0.23 (0.03)^b^	0.20 (0.03)^ab^
B. Cycliccompression-tension	Compression Stiffness (N/mm)	153 (16)^b^	206 (21)^a^	174 (9)	177 (33)
	Tensile Stiffness (N/mm)	46 (9)^b^	85 (14)^a^	56 (17)^b^	60 (7)^b^
	Neutral Zone Stiffness (N/mm)	5.78 (1.37)^b^	12.61 (2.49)^a^	8.75 (1.96)^b^	7.83 (1.35)^b^
	Neutral Zone Length (mm)	0.59 (0.13)^b^	0.35 (0.10)^a^	0.45 (0.08)	0.44 (0.13)
	Cumulative Height Loss (mm)	0.011 (0.002)	0.014 (0.002)	0.015 (0.004)	0.012 (0.003)
C. Quasi-static compression	Quasi-static Stiffness (N/mm)	63 (11) b	96 (13)^a^	74 (12)^b^	77 (7)^b^
	Cumulative Height Loss (mm)	0.11 (0.2)^b^	0.07 (0.01)^a^	0.10 (0.02)	0.09 (0.02)
D. Dynamic Compression	Dynamic Stiffness (N/mm)	243 (13)^b^	354 (20)^a^	305 (21)^ab^	291 (16)^ab^
	Cumulative Height Loss (mm)	0.11 (0.01)^b^	0.08 (0.01)^a^	0.10 (0.02)^a^	0.09 (0.02)
E. Creep	d_inf_ –d_0_ (mm)	0.056 (0.014)^b^	0.030 (0.007)^a^	0.036 (0.006)^a^	0.052 (0.015)^b^
	Cumulative Height Loss (mm)	0.17 (0.03)^b^	0.11 (0.01)^a^	0.13 (0.01)^a^	0.14 (0.02)^b^

For stage D, 1 Hz frequency was chosen as representative; cumulative height loss was recorded with respect to the end of stage A. (^a^*p* < 0.05 compared with Sham group; ^b^*p* < 0.05 compared with OVX group).
